# 2,3,4′,5-tetrahydroxystilbene-2-*O*-*β*-D-glucoside exacerbates acetaminophen-induced hepatotoxicity by inducing hepatic expression of CYP2E1, CYP3A4 and CYP1A2

**DOI:** 10.1038/s41598-017-16688-5

**Published:** 2017-11-28

**Authors:** Shangfu Xu, Jie Liu, Jingshan Shi, Zhengtao Wang, Lili Ji

**Affiliations:** 1 The MOE Key Laboratory for Standardization of Chinese Medicines, Shanghai Key Laboratory of Compound Chinese Medicines and The SATCM Key Laboratory for New Resources and Quality Evaluation of Chinese Medicines, Institute of Chinese Materia Medica, Shanghai University of Traditional Chinese Medicine, Shanghai, 201203 China; 2 Key Laboratory of Basic Pharmacology of Ministry of Education and Joint International Research Laboratory of Ethnomedicine of Ministry of Education, Zunyi Medical University, Zunyi, Guizhou 563006 China

## Abstract

Hepatotoxicity induced by medicinal herb *Polygonum multiflorum* Thunb. attracts wide attention in the world recently. 2,3,4′,5-tetrahydroxystilbene-2-*O*-*β*-D-glucoside (TSG) is a main active compound in *Polygonum multiflorum* Thunb. This study aims to observe TSG-provided the aggravation on acetaminophen (APAP)-induced hepatotoxicity in mice by inducing hepatic expression of cytochrome P450 (CYP450) enzymes. Serum alanine/aspartate aminotransferase (ALT/AST) analysis and liver histological evaluation showed that TSG (200, 400, 800 mg/kg) exacerbated the hepatotoxicity induced by sub-toxic dose of APAP (200 mg/kg) in mice, but TSG alone had no hepatotoxicity. TSG aggravated hepatic reduced glutathione (GSH) depletion and APAP-cysteine adducts (APAP-CYS) formation induced by APAP in mice. TSG increased the expression of CYP2E1, CYP3A4 and CYP1A2 both in mice and in human normal liver L-02 hepatocytes. TSG also enhanced liver catalytic activity of CYP2E1, CYP3A4 and CYP1A2 in mice. TSG induced the nuclear translocation of aryl hydrocarbon receptor (AHR) and pregnane X receptor (PXR), and TSG-provided the aggravation on APAP-induced hepatotoxicity in mice was reversed by PXR or AHR inhibitors. In summary, our results demonstrate that TSG enhances hepatic expression of CYP3A4, CYP2E1 and CYP1A2, and thus exacerbates the hepatotoxicity induced by APAP in mice. PXR and AHR both play some important roles in this process.

## Introduction

In recent years, the application of herbal medicines for the treatment of various diseases and the promotion of health is widely accepted in the world. Accordingly, herb-drug interactions are of great concern when patients concomitantly take drugs and herbs. Especially in China, the phenomenon of taking herbal medicines and Wersten medicines at the same time is very common. Many herb-drug interactions are due to the alternation of drug metabolism induced by herbs or natural products^1,2^. Liver CYP450 enzymes is the most important drug metabolizing enzymes and responsible for more than 80% of drug metabolism^3,4^.

Medicinal herb *Polygonum multiflorum* Thunb. is one of the most commonly used traditional Chinese medicines (TCMs) for restoring grey hair and anti-aging, removing toxicity for eliminating carbuncles, nourishing the liver and kidney, and it is widely used as tonic functional foods^5,6^. Recently, the safety of *Polygonum multiflorum* Thunb. has attracted wide-spread concern in the world, and its supervised usage is recommended by various countries including Canada, Britain and Australia^6,7^. A growing number of clinical studies have shown the linkage of *Polygonum multiflorum Thunb*.-containing herbal products with hepatotoxicity, but the toxicological substances and mechanisms are still unclear^8,9^. Anthraquinones (including emodin and physcion) were considered to be, at least partial, the hepatotoxic compounds^10,11^. However, some other studies showed that anthraquinones were not the culprit^12^. Moreover, it is reported that *Polygonum multiflorum* Thunb. did not cause obvious liver injury in rodents when it was given alone^13,14^. Thus it can be seen that the hepatotoxicity induced by *Polygonum multiflorum* Thunb. needs further deep investigation. A clinical report showed that only 15 cases (accounting for 9.5% of all suspected 158 cases of hepatotoxicity) were caused by the ingestion of *Polygonum multiflorum* Thunb. alone, but in 58.2% cases *Polygonum multiflorum* Thunb. was used in combination with other potential hepatotoxic medicines or prescriptions^9^. So herb-drug interactions may be a breakthrough point to study the hepatotoxicity induced by *Polygonum multiflorum* Thunb.

N-acetyl-p-aminophenol (acetaminophen or paracetamol, APAP) is widely used in clinic for its analgesic and antipyretic properties. APAP overdose will induce serious acute liver failure, and APAP-induced hepatotoxicity is reported to be the main cause for drug-induced liver injury (DILI) in the United States and the United kingdom^15,16^. N-acetyl p-benzoquinoneimine (NAPQI), a hepatotoxic metabolite of APAP, is metabolized by CYP450 enzymes in livers, specifically isoforms such as CYP2E1, CYP3A4 and CYP1A2^17,18^. The inhibition of CYP-mediated bio-activation of APAP provided by some natural products is found to contribute to their protection against APAP-induced hepatotoxicity^19–23^. However, some other compounds (such as isoniazid, caffeine, benzothiazole and ethanol) are found to aggravate APAP-induced hepatotoxicity via inducing CYP450s^24–27^.

2,3,4′,5-tetrahydroxystilbene-2-*O*-*β*-D-glucoside (TSG) is a main active compound in *Polygonum multiflorum* Thunb. with high content, and it is also a chemical marker used by the Chinese Pharmacopoeia for evaluating the quality of *Polygonum multiflorum* Thunb.^5^. TSG has been shown beneficial for human health and has various pharmacological activities such as anti-inflammatory, anti-aging, hypolipidemic, hypotensive, cardio-protective and neuro-protective effects^28–34^. A previous study showed that TSG did not produce overt hepatotoxicity *in vitro* ^35^. In this study, the aggravation of TSG on APAP-induced liver injury by inducing hepatic expression of CYP2E1, CYP3A4 and CYP1A2 was investigated.

## Results

### TSG exacerbated the hepatotoxicity induced by APAP in mice

**Figure 1:**
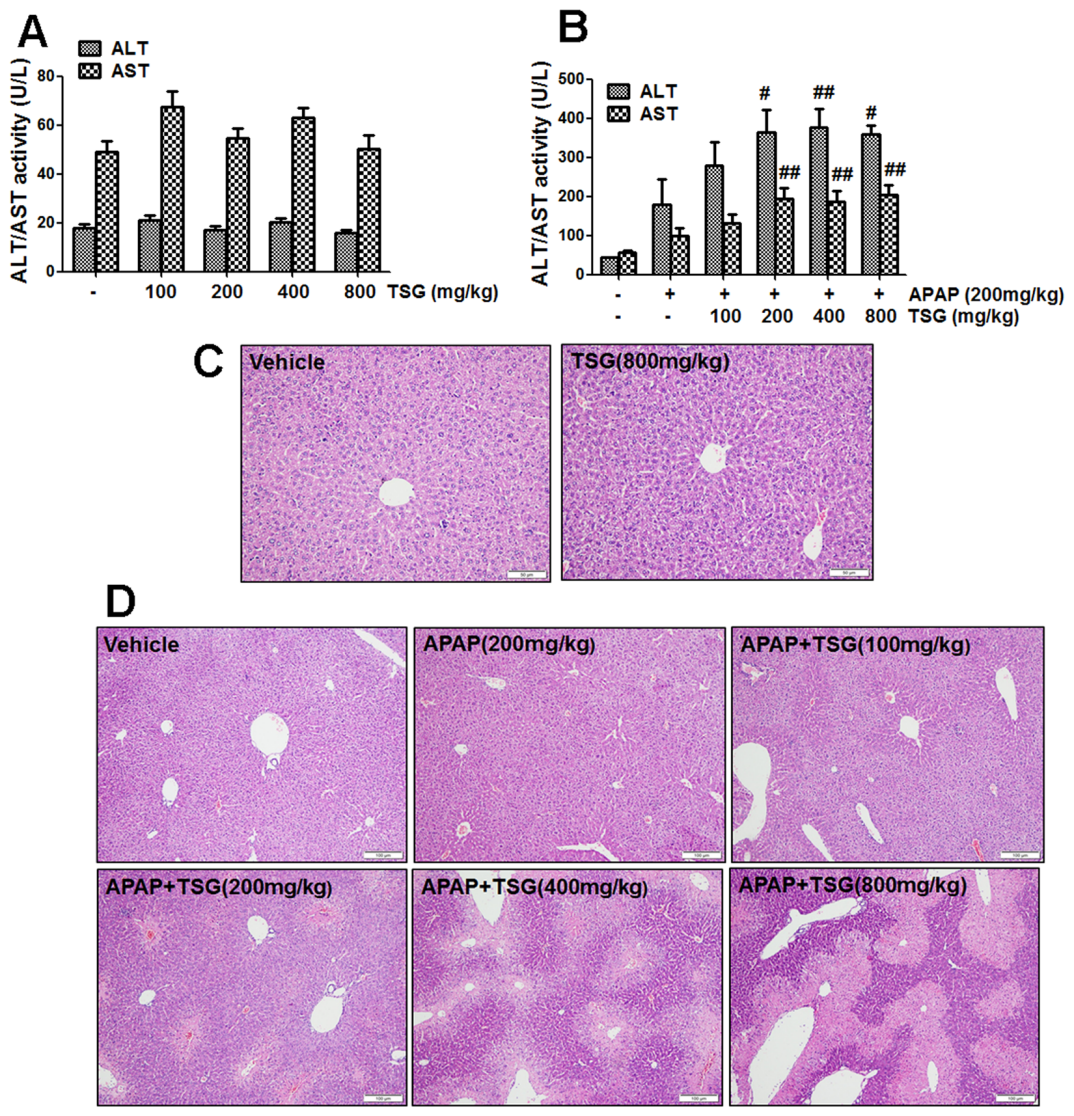
TSG exacerbated the hepatotoxicity induced by APAP in mice. (**A**) Serum ALT/AST activity was detected when TSG (100–800 mg/kg) was orally given to mice once for 12 h. (**B**) Serum ALT/AST activity was detected at 6 h after APAP (200 mg/kg) was orally given to mice once at 12 h after TSG (100–800 mg/kg) administration. (**C**–**D**) Liver histological observation. Typical images were chosen from each experimental group (original magnification ×200). Data were expressed as means ± SEM (n = 10). ^#^ *P* < 0.05, ^##^ *P* < 0.01 compared to APAP.

Data in Fig. 1A showed that TSG (100, 200, 400, 800 mg/kg) alone had no effect on serum ALT/AST activity in mice. Also, the results of liver histological evaluation showed that there were no obvious pathological changes occurred in mice treated with TSG (800 mg/kg) alone (Fig. 1C).

As shown in Fig. 1B, APAP (200 mg/kg) alone had no effect on serum ALT and AST activities in mice. The results of liver histological evaluation also did not show any obvious pathological changes in mice treated with APAP (200 mg/kg) alone (Fig. 1D). However, after mice were pre-treated with different doses of TSG (100, 200, 400, 800 mg/kg) for 12 h, TSG dose-dependently exacerbated APAP-induced liver injury evidenced by markedly increased serum ALT and AST activities (Fig. 1B). When APAP (200 mg/kg) was orally given to mice once for 6 h in TSG (100–800 mg/kg)-pre-treated mice, liver injury increased obviously in a dose-dependent manner, from cell degeneration (TSG 100 mg/kg), bridging necrosis (TSG 200 mg/kg) to apparent necrosis (TSG 400 mg/kg) and massive necrosis (TSG 800 mg/kg) (Fig. 1D).

### TSG augmented the depletion of hepatic GSH and the formation of APAP-CYS induced by APAP in mice

**Figure 2:**
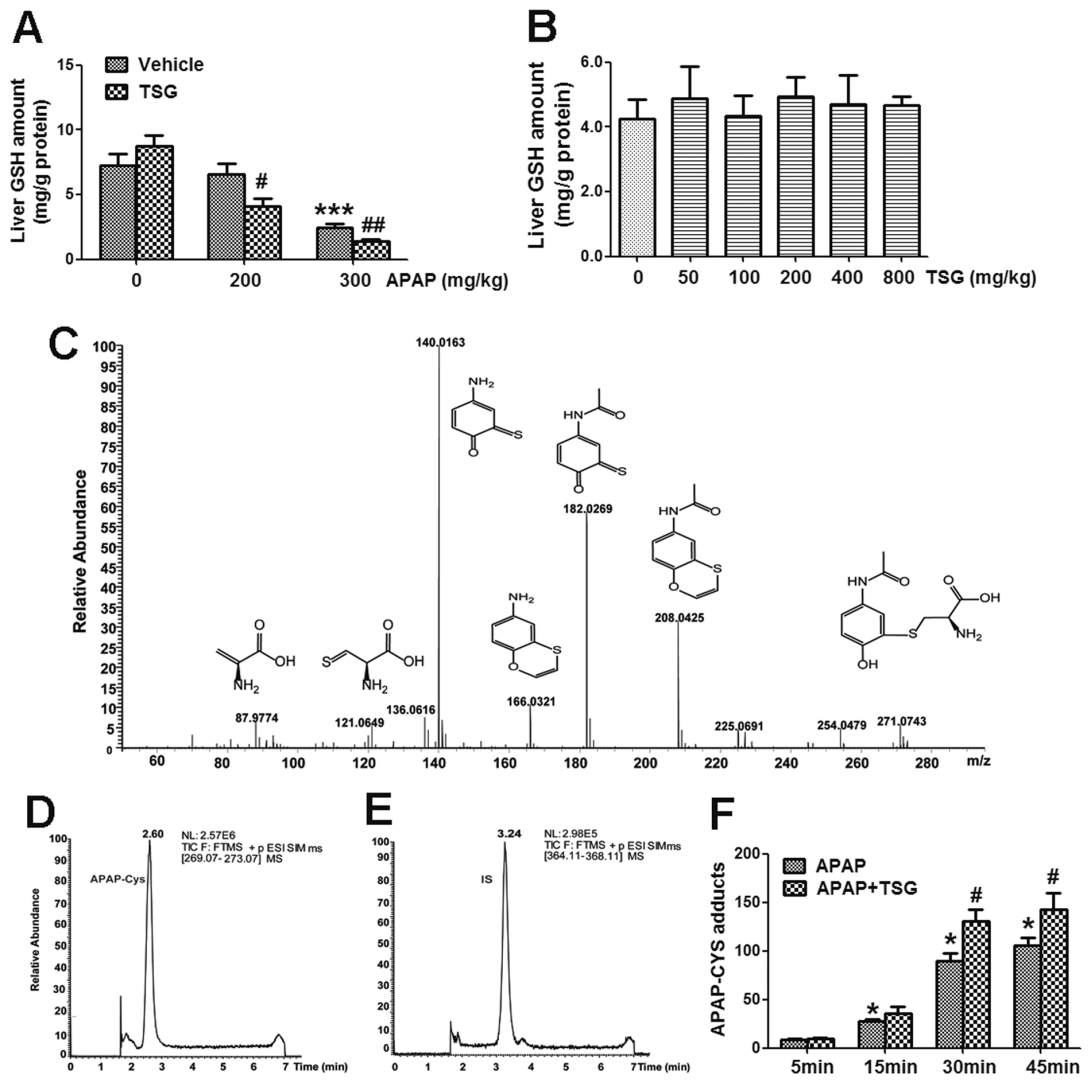
TSG exacerbated hepatic GSH depletion and APAP-CYS formation induced by APAP in mice. (**A**) Liver GSH amount was detected at 6 h after APAP (200, 300 mg/kg) was orally given to mice at 12 h after TSG (800 mg/kg) administration (n = 10). (**B**) Liver GSH amount were determined after mice were treated with different doses of TSG for 12 h (n = 10). (**C**) The ion fragments of APAP-CYS were detected by HPLC-MS/MS. (**D**) The chromatographic peak of APAP-CYS was detected by HPLC-MS/MS. (**E**) The chromatographic peak of internal standard was detected by HPLC-MS/MS. (**F**) The APAP-CYS response was detected at 5 min, 15 min, 30 min and 45 min after APAP (200 mg/kg) was orally given to mice at 12 h after TSG (800 mg/kg) administration (n = 5). Data were expressed as means ± SEM. *P < 0.05, ***P < 0.001 compared to Vehicle control; ^#^ *P* < 0.05, ^*##*^ *P < *0.01, compared to APAP.

GSH plays a crucial role in regulating the detoxification of APAP-induced liver injury by binding to NAPQI, the toxic metabolite of APAP in liver^17^. As shown in Fig. 2A, APAP (300 mg/kg) decreased hepatic GSH amount, and TSG (800 mg/kg) exacerbated this decreased GSH amount induced by APAP (300 mg/kg) when mice were pre-treated with TSG for 12 h. Hepatic GSH amount was not altered in mice treated with APAP (200 mg/kg) alone, but significantly decreased in mice treated with APAP (200 mg/kg) plus TSG (800 mg/kg) (Fig. 2A). However, there is no obvious change of hepatic GSH amount in mice treated with different doses of TSG (50–800 mg/kg) for 12 h (Fig. 2B). Also, there is no obvious change of liver GSH amount in mice treated with TSG (800 mg/kg) alone for 4 h, 8 h and 12 h (Supplementary Fig. 1).

APAP-CYS are serum biomarker of APAP exposure and hepatotoxicity, formed when NAPQI binds to cysteine resides of hepatic proteins, which indirectly reflect the formation of NAPQI after APAP metabolic activation^36,37^. Next, LC-MS/MS was used to detect serum amount of APAP-CYS in mice. As shown in Fig. 2C–F, the amount of APAP-CYS was gradually increased after APAP (200 mg/kg) was orally given to mice once at 5 min, 15 min, 30 min and 45 min. Compared to in mice treated with APAP alone, serum APAP-CYS amount was obviously elevated in APAP-treated mice with TSG (800 mg/kg) pre-administration, especially at 30 min and 45 min (Fig. 2C–F).

### TSG increased hepatic expression of CYP1A2, CYP2E1 and CYP3A4 in mice

**Figure 3:**
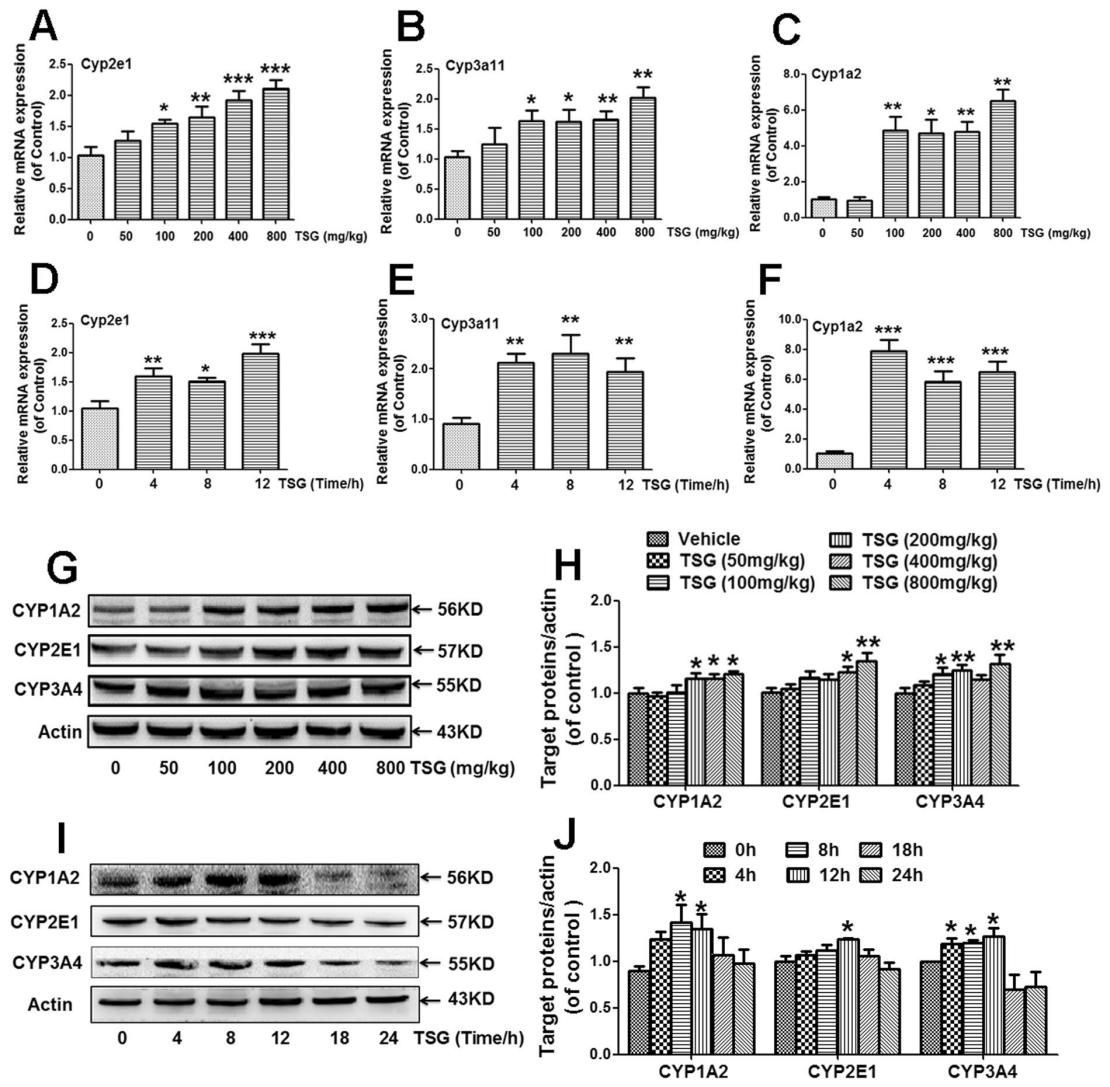
TSG increased hepatic expression of CYP1A2, CYP2E1 and CYP3A4 in mice. (**A**–**C**) Hepatic mRNA expression of Cyp1a2, Cyp2e1 and Cyp3a11 (human CYP3A4) was detected at 12 h after mice were treated with different doses of TSG (50–800 mg/kg). (**D**–**F**) Hepatic mRNA expression of Cyp1a2, Cyp2e1 and Cyp3a11 was detected at 4 h, 8 h or 12 h after TSG (800 mg/kg) administration. (**G**–**H**) Hepatic protein expression of CYP1A2, CYP2E1 and CYP3A4 was detected at 12 h after mice were treated with different doses of TSG (50–800 mg/kg). The results represent six independent experiments. (**I**–**J**) Hepatic protein expression of CYP1A2, CYP2E1 and CYP3A4 was detected at 4 h, 8 h or 12 h after TSG (800 mg/kg) administration. The results represent six independent experiments. Data were expressed as means ± SEM (n = 6). *P < 0.05, **P < 0.01, ***P < 0.001 compared to Vehicle control.

Liver mRNA expression of Cyp1a2, Cyp3a11 (human CYP3A4) and Cyp2e1 was increased in mice treated with different doses of TSG (100, 200, 400, 800 mg/kg) for 12 h (Fig. 3A–C). Additionally, when mice were treated with TSG (800 mg/kg) for different times, liver mRNA expression of Cyp1a2, Cyp2e1 and Cyp3a11 was increased in mice treated with TSG (800 mg/kg) for 4 h, 8 h, and 12 h (Fig. 3D–F).

Next, western-blot analysis was used to further confirm the increased hepatic CYP2E1, CYP3A4 and CYP1A2 expression induced by TSG in mice. TSG (200, 400, 800 mg/kg) enhanced hepatic CYP1A2 protein expression when mice were treated with different doses of TSG for 12 h (Fig. 3G,H). When mice were treated with TSG (800 mg/kg) for different times, TSG increased hepatic CYP1A2 expression after treated mice for 8 h and 12 h (Fig. 3I,J). TSG (400, 800 mg/kg) enhanced hepatic CYP2E1 protein expression when mice were treated with different doses of TSG for 12 h (Fig. 3G,H). When mice were treated with TSG (800 mg/kg) for different times, TSG only increased hepatic CYP2E1 expression after treated mice for 12 h (Fig. 3I,J). TSG (100, 200, 800 mg/kg) enhanced hepatic CYP3A4 protein expression when mice were treated with different doses of TSG for 12 h (Fig. 3G,H). When mice were treated with TSG (800 mg/kg) for different times, TSG increased hepatic CYP3A4 expression after treated mice for 4 h, 8 h and 12 h (Fig. 3I,J).

### TSG enhanced liver catalytic activity of CYP1A2, CYP2E1 and CYP3A4 in mice

**Figure 4:**
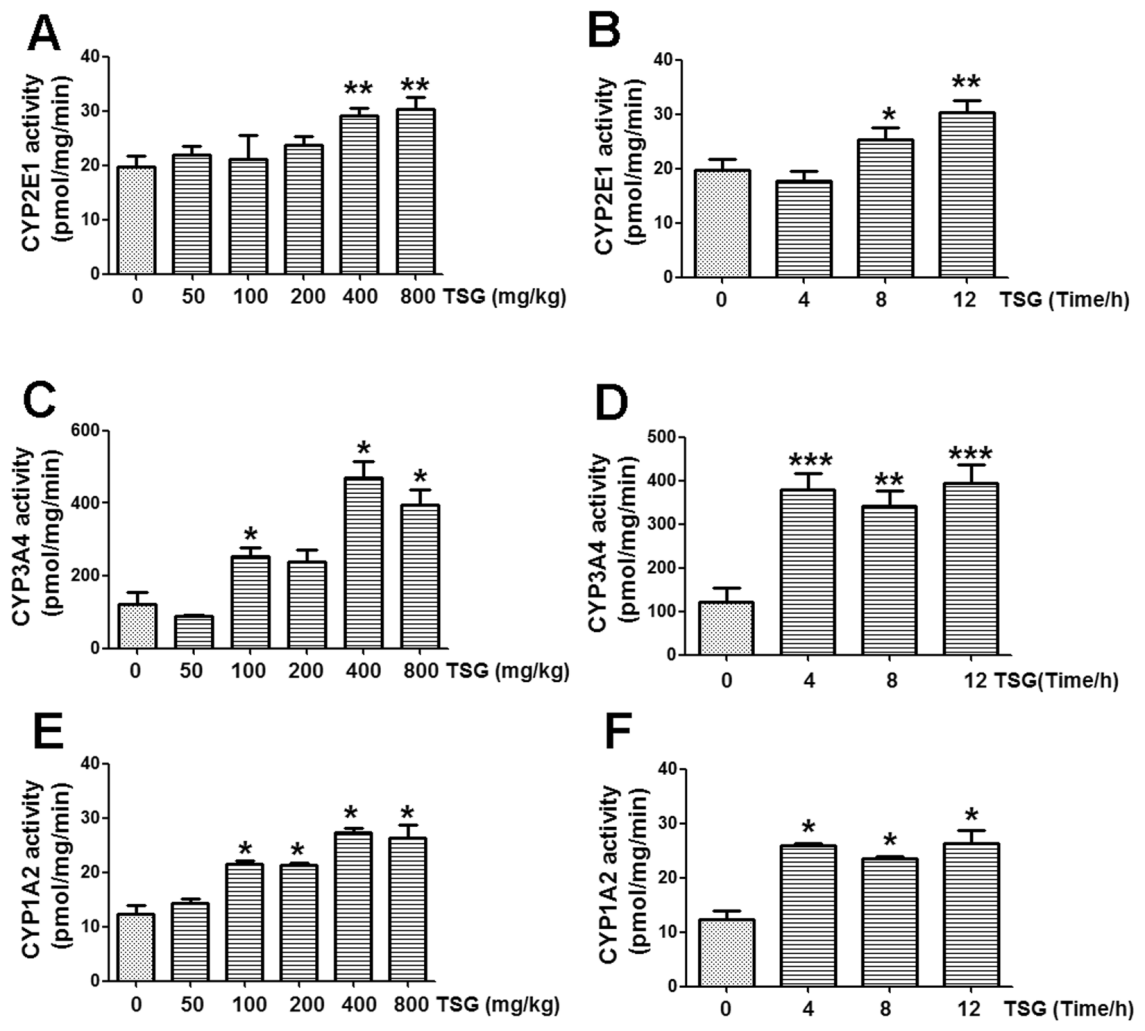
TSG increased liver catalytic activity of CYP1A2, CYP2E1 and CYP3A4 in mice. (**A**) Hepatic CYP2E1 catalytic activity was detected at 12 h after mice were treated with different doses of TSG (50–800 mg/kg). (**B**) Hepatic CYP2E1 catalytic activity was detected at 4 h, 8 h or 12 h after TSG (800 mg/kg) administration. (**C**) Hepatic CYP3A4 catalytic activity was detected at 12 h after mice were treated with different doses of TSG (50–800 mg/kg). (**D**) Hepatic CYP3A4 catalytic activity was detected at 4 h, 8 h or 12 h after TSG (800 mg/kg) administration. (**E**) Hepatic CYP1A2 catalytic activity was detected at 12 h after mice were treated with different doses of TSG (50–800 mg/kg). (**F**) Hepatic CYP1A2 catalytic activity was detected at 4 h, 8 h or 12 h after TSG (800 mg/kg) administration. Data were expressed as means ± SEM (n = 4–6). *P < 0.05, **P < 0.01, ***P < 0.001 compared to Vehicle control.

TSG (400, 800 mg/kg) enhanced liver CYP2E1 catalytic activity when mice were treated with different doses of TSG for 12 h (Fig. 4A). When mice were treated with TSG (800 mg/kg) for different times, TSG increased liver CYP2E1 catalytic activity after treated mice for 8 h and 12 h (Fig. 4B).

TSG (100, 400, 800 mg/kg) enhanced liver CYP3A4 catalytic activity when mice were treated with different doses of TSG for 12 h (Fig. 4C). When mice were treated with TSG (800 mg/kg) for different times, TSG increased liver CYP3A4 catalytic activity after treated mice for 4 h, 8 h and 12 h (Fig. 4D).

TSG (100, 200, 400, 800 mg/kg) enhanced liver CYP1A2 catalytic activity when mice were treated with different doses of TSG for 12 h (Fig. 4E). When mice were treated with TSG (800 mg/kg) for different times, TSG increased liver CYP1A2 catalytic activity after treated mice for 4 h, 8 h and 12 h (Fig. 4F).

### TSG exacerbated APAP-induced hepatotoxicity and increased CYP1A2, CYP2E1, CYP3A4 expression *in vitro*

**Figure 5:**
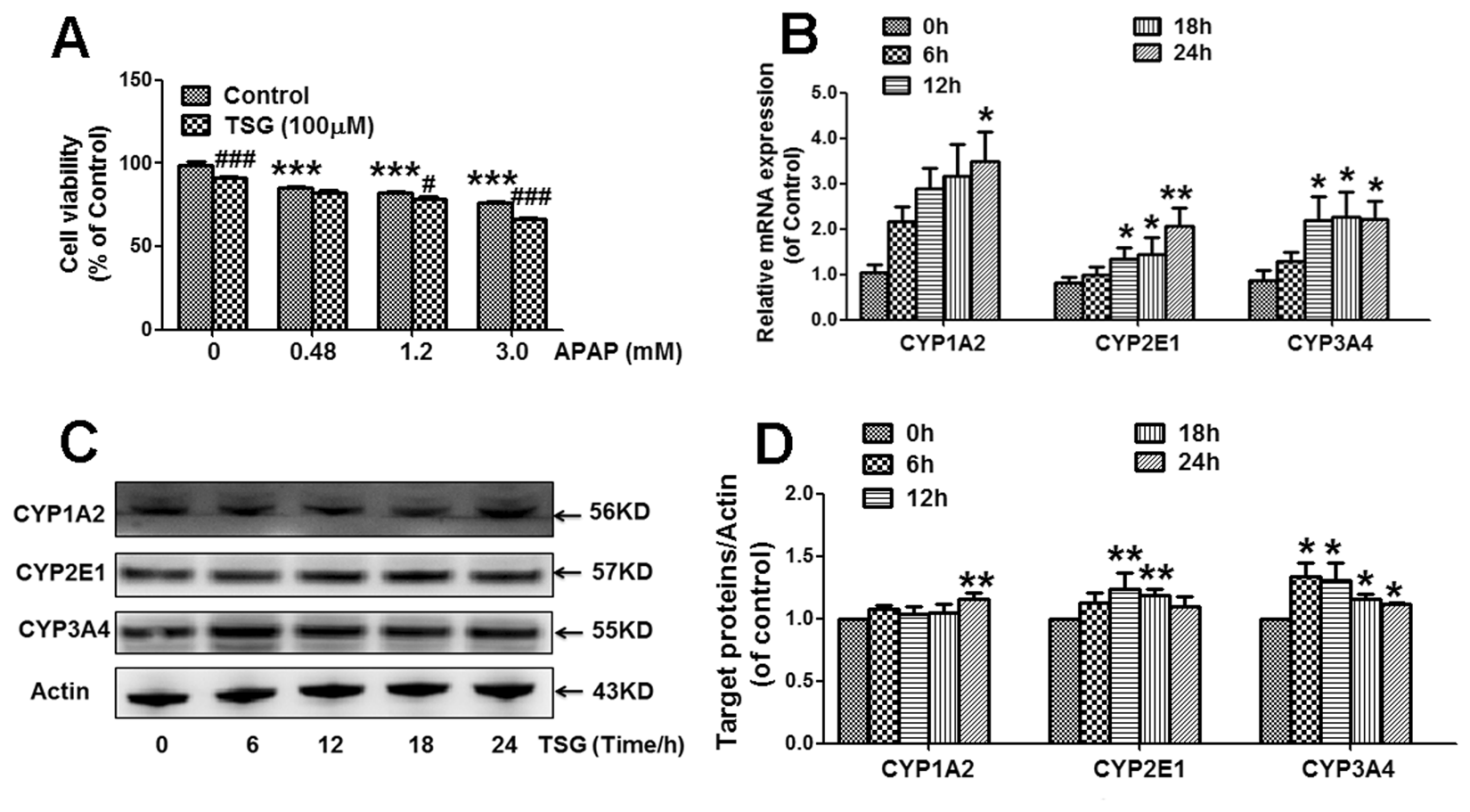
Effects of TSG on CYP1A2, CYP3A4 and CYP2E1 expression *in vitro*. (**A**) L-02 cells were pre-incubated with TSG (100 μM) for 24 h, and then incubated with APAP (0.48, 1.2, 3.0 mM) for 48 h. After treatment, survival cells were determined (n = 5). (**B**) Cellular mRNA expression of CYP1A2, CYP2E1 and CYP3A4 was detected when cells were treated with TSG (100 μM) for 6 h, 12 h, 18 h and 24 h, respectively (n = 3). (**C**–**D**) Cellular protein expression of CYP1A2, CYP2E1 and CYP3A4 was detected when cells were treated with TSG (100 μM) for 6 h, 12 h, 18 h and 24 h, respectively. The results represent at least three independent experiments (n = 3 for CYP3A4; n = 4 for CYP2E1; n = 6 for CYP1A2). Data were expressed as means ± SEM. *P < 0.05, **P < 0.01, ***P < 0.001 compared to Vehicle control; ^#^ *P* < 0.05, ^###^ *P* < 0.001 compared to APAP.

TSG (100 μM) exacerbated the cytotoxicity induced by different concentrations of APAP (0.48, 1.2, 3.0 mM) in L-02 hepatocytes (Fig. 5A). Cellular mRNA expression of CYP1A2 was increased when L-02 cells were incubated with TSG (100 μM) for 24 h (Fig. 5B). Cellular mRNA expression of CYP2E1 was increased when L-02 cells were incubated with TSG (100 μM) for 12 h, 18 h and 24 h (Fig. 5B). Cellular mRNA expression of CYP3A4 was increased when L-02 cells were incubated with TSG (100 μM) for 12 h, 18 h and 24 h (Fig. 5B).

Next, TSG-induced the increase in the protein expression of cellular CYP1A2, CYP2E1 and CYP3A4 was detected. Cellular protein expression of CYP1A2 was enhanced when L-02 cells were incubated with TSG (100 μM) for 24 h (Fig. 5C,D). Cellular protein expression of CYP2E1 was enhanced when L-02 cells were incubated with TSG (100 μM) for 12 h and 18 h, (Fig. 5C,D). Cellular protein expression of CYP3A4 was enhanced when L-02 cells were incubated with TSG (100 μM) for 6 h, 12 h, 18 h and 24 h (Fig. 5C,D).

### TSG induced the nuclear translocation of AHR and PXR *in vitro* and *in vivo*

**Figure 6:**
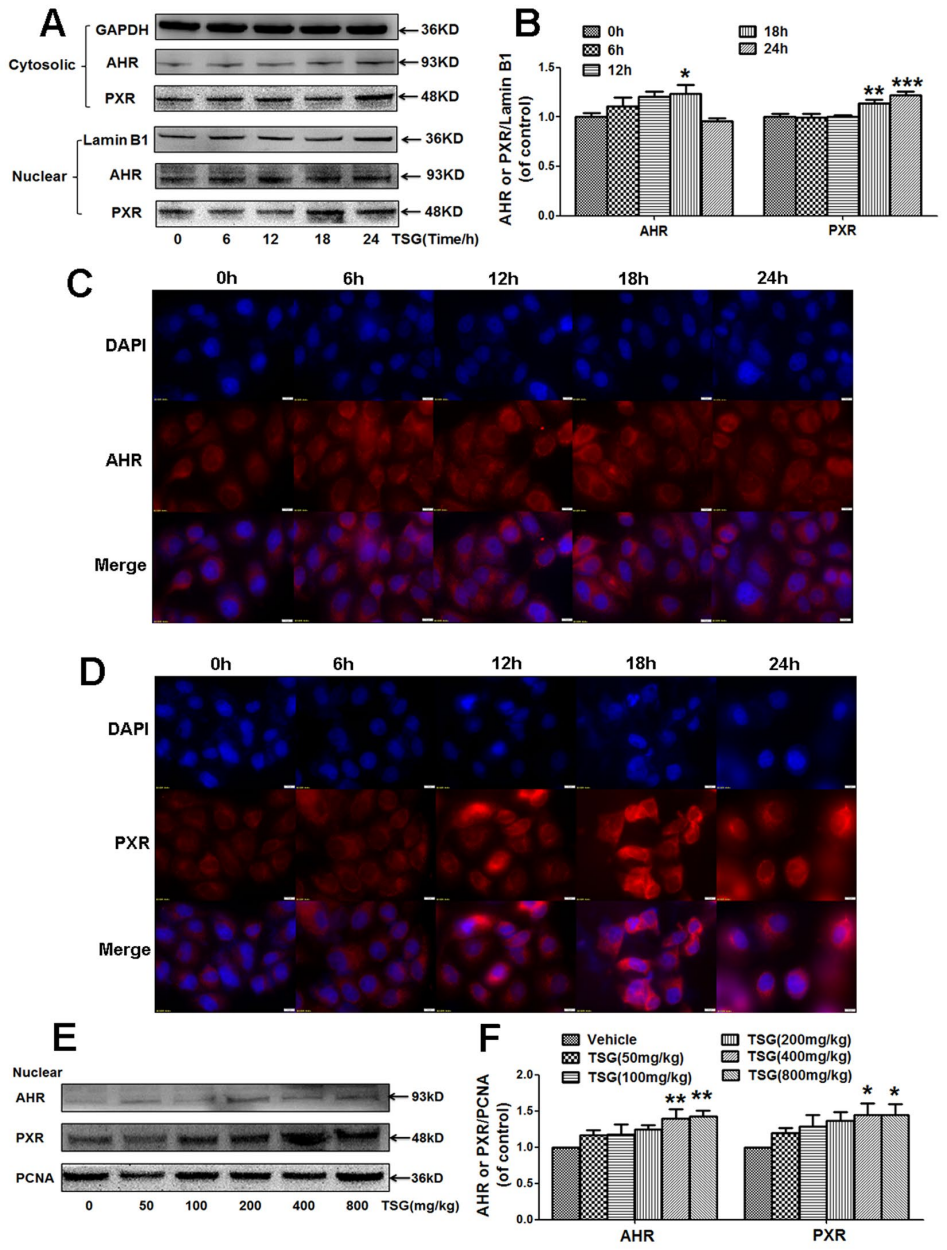
TSG increased the nuclear translocation of AHR and PXR both *in vitro* and *in vivo*. (**A**–**B**) L-02 cells were incubated with TSG (100 μM) for 6 h, 12 h, 18 h and 24 h. Cellular protein expression of AHR and PXR was detected by western-blotting, and GAPDH and Lamin B1 were used as loading controls. The results represent four independent experiments. (**C**) L-02 cells were incubated with TSG (100 μM) for 6 h, 12 h, 18 h and 24 h, and then AHR localization was observed under an inverted fluorescence microscope (magnification ×600). Results represent three independent experiments. (**D**) L-02 cells were incubated with TSG (100 μM) for 6 h, 12 h, 18 h and 24 h, and then PXR localization was observed under an inverted fluorescence microscope (magnification ×600). Results represent three independent experiments. (**E**–**F**) Mice were treated with TSG (50–800 mg/kg) for 12 h. After treatment, hepatic nuclear protein expression of AHR and PXR were detected by western-blotting, and PCNA was used as a loading control. The results represent three independent experiments. Data were expressed as means ± SEM. *P < 0.05, **P < 0.01, ***P < 0.001 compared to Vehicle control.

AHR and PXR are reported to regulate the expression of CYP1A2 and CYP3A4^38^. Next, whether TSG will induce the nuclear translocation of AHR or PXR in hepatocytes was observed. As shown in Fig. 6A,B TSG (100 μM) induced AHR nuclear translocation in L-02 cells when cells were incubated with TSG for 18 h. Furthermore, TSG (100 μM) induced PXR nuclear translocation in L-02 cells when cells were incubated with TSG for both 18 h and 24 h. The results of immunofluorescence staining assay showed that the nuclear translocation of AHR was seen when cells were incubated with TSG (100 μM) for 18 h or 24 h (Fig. 6C), and the nuclear translocation of PXR was seen when cells were incubated with TSG (100 μM) for 12 h, 18 h or 24 h (Fig. 6D). Additionally, the nuclear translocation of AHR and PXR was also observed in liver tissues from mice treated with TSG (400, 800 mg/kg) for 12 h (Fig. 6E,F).

### Effects of AHR or PXR inhibitor on the liver injury induced by APAP with TSG pre-administration in mice

**Figure 7:**
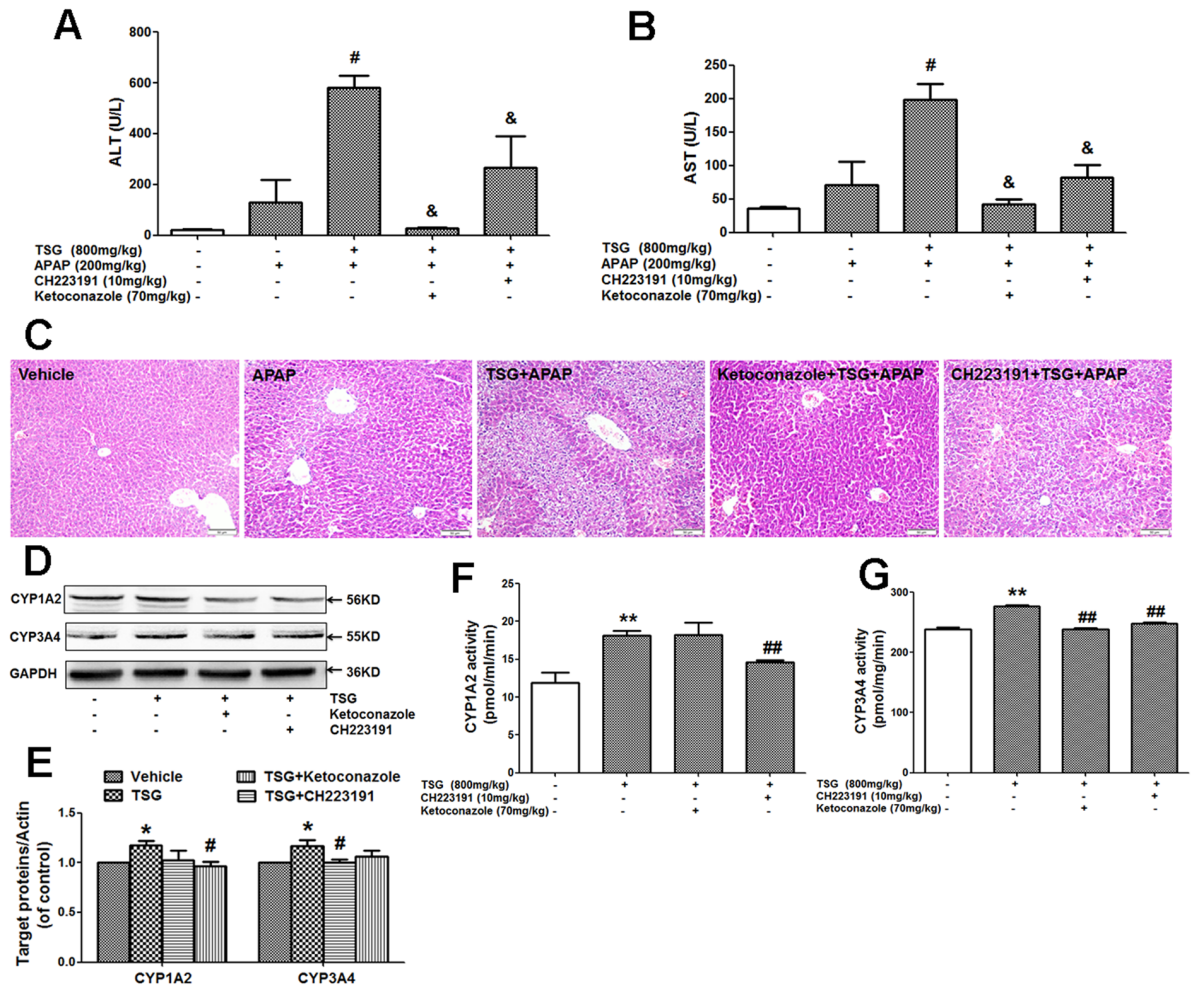
Effects of AHR or PXR inhibitor on the liver injury induced by APAP with TSG pre-administration in mice. (**A**–**B**) Serum ALT/AST activity was detected when mice were orally given with APAP (200 mg/kg) for 6 h with TSG (800 mg/kg) administration for 12 h after pre-administrated with or without ketoconazole (70 mg/kg) or CH223191 (10 mg/kg) for 3 d (Once a day) (n = 7). (**C**) Liver histological observation. Typical images were chosen from each experimental group (original magnification ×200). (**D**–**E**) Hepatic protein expression of CYP1A2 and CYP3A4 was analyzed at 12 h after TSG (800 mg/kg) administration when mice were pre-administrated with or without ketoconazole (70 mg/kg) or CH223191 (10 mg/kg) for 3 days (Once a day). The results represent five independent experiments. (**E**) Hepatic catalytic activity of CYP1A2 and CYP3A4 was analyzed at 12 h after TSG (800 mg/kg) administration when mice were pre-administrated with or without ketoconazole (70 mg/kg) or CH223191 (10 mg/kg) for 3 days (Once a day) (n = 4). Data were expressed as means ± SEM. *P < 0.05, **P < 0.01 compared to Vehicle control; ^#^ *P* < 0.05, ^##^ *P* < 0.01 compared to APAP or TSG; ^&^ *P* < 0.05 compared to TSG plus APAP.

Ketoconazole and CH223191 are specific inhibitors for AhR and PXR, respectively^39,40^. As shown in Fig. 7A–C, TSG (800 mg/kg) exacerbated APAP (200 mg/kg)-induced liver injury in mice evidenced by the elevated serum ALT/AST activity and increased liver lesions from liver histological evaluation. However, after mice were pre-treated with ketoconazole or CH223191, the exacerbated effect provided by TSG on APAP-induced hepatotoxicity was obviously reduced, as evidenced by the results from serum ALT/AST activity and liver histological evaluation (Fig. 7A–C). TSG enhanced hepatic expression of CYP1A2 and CYP3A4 in mice (Fig. 7D,E). However, after mice were pre-treated with ketoconazole or CH223191, TSG-induced the increased CYP1A2 and CYP3A4 protein expression was reduced. TSG-induced the increase in liver CYP1A2 activity in mice was abrogated by CH223191 (Fig. 7F). Additionally, TSG-induced the increase in liver CYP3A4 activity in mice was abrogated by both ketoconazole and CH223191 (Fig. 7G). However, TSG (800 mg/kg) plus ketoconazole or CH223191 had no effect on serum ALT/AST activity in mice (Supplementary Fig. 2).

## Discussion

*Polygonum multiflorum* Thunb. is an ingredient in many medicines and prescriptions, and has been widely used to treat a variety of diseases^6^. However, recent reports demonstrated that it could lead to liver injury and even death in clinic^7,8,41^, which had aroused wide concern in the world. TSG is the main compound with highest content in *Polygonum multiflorum* Thunb., and the content of TSG shall be more than 1% in Polygoni Multiflori Radix and more than 0.7% in Polygoni Multiflori Radix Praeparata^5^. A previous study showed that TSG had no hepatotoxicity *in vitro* ^35^. Our results in this study showed that TSG had no obvious hepatotoxicity in mice. Thus, TSG did not produce overt hepatotoxicity both *in vivo* and *in vitro*, and it is not the component with direct hepatotoxicity in *Polygonum multiflorum* Thunb.

Recent studies have shown the idiosyncratic hepatotoxicity induced by *Polygonum multiflorum* Thunb., and TSG might induce immunological idiosyncratic hepatotoxicity^14,42^. In this study, TSG (200–800 mg/kg) augmented the liver injury induced by sub-toxic dose of APAP (200 mg/kg), as evidenced by the elevated serum ALT/AST activity and the increased liver lesions from liver histological evaluation. Additionally, TSG also increased APAP-induced cytotoxicity in human normal liver L-02 cells. All these above results evidenced the aggravation of TSG on the liver injury induced by APAP. Also, the dose of TSG-provided aggravation on APAP-induced liver injury is at least 200 mg/kg, which is high and cannot be reached when *Polygonum multiflorum* Thunb. was used under the normal and recommended dose recorded in Chinese Pharmacopoeia. However, *Polygonum multiflorum* Thunb. is often taken at an overdose as a tonic functional food and health natural product. Especially in southern China, a lot of people in the life like to take it soaked in wine, in boil soup or porridge, or even eat it as powder. Additionally, as recorded in the textbook of “Chinese Materia Medica”, the usage of 10–30 g Polygoni Multiflori Radix or Polygoni Multiflori Radix Praeparata was recommended^43^. The content of TSG in *Polygonum multiflorum* Thunb. is generally higher than 1%, even close to 10%^44,45^. Therefore, the dose range of TSG (50–400 mg/kg) in this study falls within the potential individual concentration of *Polygonum multiflorum* Thunb. So, our results pointed out the potential risk of the overdose of *Polygonum multiflorum* Thunb.

NAPQI, the toxic metabolite of APAP in liver, is highly reactive and is primarily responsible for APAP-induced hepatotoxicity. The detoxification of NAPQI occurs through its binding to GSH. NAPQI also binds to sulfhydryl groups on cysteine residues of hepatic proteins and forms APAP-CYS, which are released into circulatory system following liver injury and become measurable^17,36,46,47^. The elevated APAP-CYS amount indirectly reflects the increased liver metabolic formation of APAP into NAPQI^36^. In this study, TSG increased the depletion of hepatic GSH and enhanced the formation of APAP-CYS induced by APAP in mice. These results imply that TSG may augment APAP-induced liver injury by enhancing the metabolic activation of APAP into its toxic metabolite NAPQI.

CYP450 enzymes are responsible for metabolizing APAP into its reactive metabolite NAPQI, and of which CYP1A2, CYP2E1 and CYP3A4 are the main CYP450 enzymes for the metabolic activation of APAP in liver^17,48,49^. Previous studies showed that some chemical agents were reported to exacerbate APAP-induced liver injury by enhancing its liver metabolism via inducing the expression of CYP450 enzymes^24–27^. In this study, when mice were treated with TSG for 12 h, TSG increased hepatic expression of CYP2E1, CYP1A2 and CYP3A4, and also elevated liver catalytic activity of CYP2E1, CYP1A2 and CYP3A4. Moreover, when mice were treated with TSG for 5 consecutive days, TSG also enhanced hepatic expression of CYP2E1, CYP1A2 and CYP3A4. All these results indicate that TSG-induced the increased expression and catalytic activity of CYP1A2, CYP2E1 and CYP3A4 may contribute to its augmentation on APAP-induced hepatotoxicity via elevating its metabolic activation in liver.

AHR and PXR are found to be responsible for regulating the transcriptional expression of CYP1A2 and CYP3A4^38,50^. In this study, TSG promoted the nuclear translocation of AHR and PXR both *in vivo* and *in vitro*, which may contribute to the increased expression of CYP1A2 and CYP3A4 induced by TSG. Furthermore, TSG-induced the exacerbation on APAP-induced hepatotoxicity markedly reduced in mice when mice were pre-treated with specific inhibitors of AHR or PXR. Additionally, AHR or PXR inhibitors reduced the expression and activity of hepatic CYP1A2 and CYP3A4 in mice. These results indicate that AHR and PXR both play important roles in regulating TSG-provided the exacerbation on APAP-induced liver injury.

In conclusion, our results found that TSG exacerbated APAP-induced liver injury at the first time. Next, this study showed that TSG augmented APAP-induced liver injury by increasing the expression and catalytic activation of liver CYP3A4, CYP1A2 and CYP2E1. Furthermore, PXR and AHR both played some important roles in regulating TSG-provided the aggravation on APAP-induced hepatotoxicity in mice. This study reminds people shall pay high attention to the potential hepatotoxicity due to herb-drug interaction when *Polygonum multiflorum* Thunb. was used in combination with other hepatotoxic drugs.

## Methods

### Materials

TSG was purchased from Nanjing Zelang Biological Technology Co., Ltd. (Nanjing, China), with the purity over 99%. APAP was purchased from Sigma Chemical Co. (St. Louis, MO). Ketoconazole was purchased from National Institutes for Food and Drug Control of China (Beijing, China). CH223191 was purchased from Selleck Chemicals (Houston, TX). Kits for detecting ALT/AST activity and GSH amount were purchased from Nanjing Jiancheng Bioengineering Institute (Nanjing, China). Kits for detecting CYP1A2, CYP2E1 and CYP3A4 catalytic activity were purchased from Shanghai GENMED gene Pharmaceutical Technology Co. Ltd (Shanghai, China). RPMI1640 and fetal bovine serum (FBS) were purchased from Life Technology (Carlsbad, CA). NE-PER Nuclear and Cytoplasmic Extraction Reagents was purchased from ThermoFisher Scientific (Waltham, MA). BCA Protein Assay Kit was purchased from Shanghai beyotime biological technology Co. Ltd (Shanghai, China). PrimeScript RT Master Mix and SYBR Premix Ex Taq were obtained from Takara (Shiga, Japan). Antibodies including anti-CYP1A2, -CYP2E1, -CYP3A4, -AHR and -PXR were all purchased from Beijing biosynthesis biotechnology CO., LTD (Beijing, China) (all 1:1000 dilution). Antibodies including anti-Actin, -PCNA and -Lamin B1 were purchased from Proteintech Group, Inc. (Wuhan, China) (all 1:5000 dilution). Peroxidase-conjugated goat anti-rabbit immunoglobulin G (IgG) (H + L) and anti-mouse IgG (H + L) were purchased from Shanghai beyotime biological technology Co. Ltd (Shanghai, China) (all 1:1000 dilution). Mouse Anti-rabbit IgG/PE-CY5 was purchased from Beijing biosynthesis biotechnology CO., LTD (Beijing, China) (1:1000 dilution). Other reagents unless indicated were purchased from Sigma Chemical Co. (St. Louis, MO).

### Animals and treatments

Specific pathogen-free male C57BL/6 mice (18–22 g) were purchased from Shanghai Laboratory Animal Center of Chinese Academy of Science (Shanghai, China) and Experimental Animal Center of Third Military Medical University (Chongqing, China). The animal facilities were controlled with 22 ± 1 °C, 50 ± 2% humidity and a 12 h: 12 h light: dark cycle. Mice were supplied with standard laboratory diet and water and acclimatized for one week before experiments. All the experimental procedures were performed in accordance with Chinese Guidelines of Animal Care and Welfare, and the present study was approved by the Experimental Animal Ethical Committee of Shanghai University of Traditional Chinese Medicine (Shanghai, China) and Zunyi Medical University (Zunyi, China).

For analyzing the effects of TSG on APAP-induced hepatotoxicity, two batches of mice experiments were implemented. In the first experiment, sixty mice were randomly divided into 6 groups, and each group contains 10 mice. (1) Vehicle control, (2) APAP (200 mg/kg), (3) TSG (100 mg/kg) + APAP, (4) TSG (200 mg/kg) + APAP, (5) TSG (400 mg/kg) + APAP, (6) TSG (800 mg/kg) + APAP. Mice were orally given with a single dose of APAP (200 mg/kg, intragastric administration, i.g.) after pre-administrated with different doses of TSG (i.g.) once for 12 h. In the second experiment, sixty mice were randomly divided into 6 groups, and each group contains 10 mice. (1) Vehicle control, (2) TSG (800 mg/kg), (3) APAP (200 mg/kg), (4) APAP (300 mg/kg), (5) TSG + APAP (200 mg/kg), (6) TSG + APAP (300 mg/kg). Mice were orally given with a single dose of APAP (200 or 300 mg/kg, i.g.) after pre-administrated with or without TSG (800 mg/kg, i.g.) once for 12 h. After treatment, the plasma and liver tissues of mice were collected.

For detecting the production of APAP-CYS, forty mice were randomly divided into 8 groups, and each group contains 5 mice. (1) APAP (5 min), (2) APAP (15 min), (3) APAP (30 min), (4) APAP (45 min), (5) TSG + APAP (5 min), (6) TSG + APAP (15 min), (7) TSG + APAP (30 min), (8) TSG + APAP (45 min). Mice were orally given with a single dose of APAP (200 mg/kg, i.g.) for 5 min, 15 min, 30 min and 45 min after pre-administrated with or without TSG (800 mg/kg, i.g.) once for 12 h. After treatment, the plasma of mice was collected.

For analyzing the effects of TSG on the expression and activity of CYP1A2, CYP2E1 and CYP3A4, two batches of mice experiments were implemented. In the first experiment, sixty mice were randomly divided into 6 groups, and each group contains 10 mice. (1) Vehicle control, (2) TSG (50 mg/kg), (3) TSG (100 mg/kg), (4) TSG (200 mg/kg), (5) TSG (400 mg/kg), (6) TSG (800 mg/kg). Mice were orally given with different doses of TSG (i.g.) once for 12 h. In the second experiment, forty mice were randomly divided into 4 groups, and each group contains 10 mice. (1) Vehicle control, (2) TSG (4 h), (3) TSG (8 h), (4) TSG (12 h). Mice were orally given with TSG (800 mg/kg, i.g.) once for 4 h, 8 h and 12 h. After treatment, liver tissues of mice were collected.

For observing the effects of AHR and PXR inhibitors, two batches of mice experiments were implemented. In the first experiment, fifty mice were randomly divided into 5 groups, and each group contains 10 mice. (1) Vehicle control, (2) APAP, (3) TSG + APAP, (4) Ketoconazole + TSG + APAP, (5) CH223191 + TSG + APAP. Mice were orally given with TSG (800 mg/kg, i.g.) after pre-administrated with or without ketoconazole (70 mg/kg, i.g.) or CH223191 (10 mg/kg, i.g.) for 3 days (Once a day), and then given with APAP (200 mg/kg, i.g.) for 6 h. In the second experiment, twenty mice were randomly divided into 4 groups, and each group contains 5 mice. (1) Vehicle control, (2) TSG, (3) Ketoconazole + TSG, (4) CH223191 + TSG. Mice were orally given with TSG (800 mg/kg, i.g.) for 12 h after pre-administrated with or without ketoconazole (70 mg/kg, i.g.) or CH223191 (10 mg/kg, i.g.) for 3 days (Once a day). After treatment, the plasma and liver tissues of mice were collected.

### Analysis of serum ALT/AST activities

The blood samples obtained were kept at room temperature for 1 h. Serum was then collected after centrifugation at 840 × g for 15 min. Serum ALT and AST activities were measured with kits according to the manufacturer’s instructions.

### Liver histological observation

Slices of mice livers were fixed in 10% neutral formalin (PBS dilution) for at least 24 h and subjected to standard histological procedures and embedded in paraffin. Samples were sectioned at a thickness of 5 μm, stained with hematoxylin and eosin (H&E), and evaluated for histopathological lesions randomly by chosen histological fields at 200× magnification under a light microscope (Olympus, Japan).

### Analysis of liver GSH amount

Liver tissues were homogenized with normal saline (1:9) under the condition of ice baths. Supernatant was then collected after centrifugation at 3000 × g for 10 min. Protein concentration of the supernatant was detected by BCA Kits, and liver GSH amounts (supernatant) were determined with kits according to the manufacturer’s instructions. The detection principle was that yellow compounds produced by the reaction of 5, 5′-dithiobis (2-nitrobenzoic acid) (DTNB) with sulfhydryl compounds. The amount of GSH was calculated based on tissue protein concentration and expressed as mg GSH per g protein.

### APAP-CYS adducts detection

Serum APAP-CYS adducts was detected as described in previous published paper^36^. Serum samples were frozen and stored at −80 °C until analysis, which was conducted with a LC-MS/MS method by Q Exactive HF Hybrid Quadrupole-Orbitrap MS (Thermo Fisher, USA). The internal standard omeprazole was added to all assay tubes followed by acetonitrile to precipitate the enzyme in serum samples. Enzyme was then pelleted using equal volumes of methanol: water. After centrifugation, the supernatant was reconstituted using 200 μL of 0.1% formic acid in water. The supernatant was then passed through an Ultrafree-MC centrifugal filter device and transferred into autosampler vials for LC-MS/MS analysis.

### CYP450 catalytic activity assay

CYP1A2, CYP2E1 and CYP3A4 catalytic activity were detected by using GENMED CYP1A2, CYP2E1 and CYP3A4 Kits. Liver microsomes were prepared by differential centrifugation methods. Protein concentrations of microsomes were measured by using BCA protein assay kits, and all the samples in the same experiment were normalized to the equal protein concentration (1 μg/μl). The activity of 7-methoxyresorufin-O-demethylase (MROD) is the diagnostic marker of CYP1A2. The activity of CYP1A2 was determined by fluorescence value of resorufin conversed from 7-methoxyresorufin by MROD. The activity of 7-methoxy-4-trifluoromethylcoumarin dealkylation (MFCD) is the diagnostic marker of CYP2E1. The activity of CYP2E1 was determined by fluorescence value of 7-hydroxy-4-trifluoromethylcoumarin (HFC) conversed from 7-methoxy-4-trifluoromethyl-coumarin (MFC) by MFCD. The activity of 7-benzyloxy-4trifluoromethylcoumarin dealkylation (BFCD) is the diagnostic marker of CYP3A4. The activity of CYP3A4 was determined by fluorescence value of HFC conversed from 7-benzyloxy-4-trifluoromethyl-coumarin by BFCD. The activity of CYP1A2, CYP2E1 and CYP3A4 were measured with kits according to the manufacturer’s instructions.

### Cell culture

The L-02 cell line was derived from an adult human normal liver (Cell Bank, Type Culture Collection of Chinese Academy of Sciences, Shanghai). L-02 cells were cultured in RPMI1640 supplemented with 10% [v/v] fetal bovine serum, 100 U/ml penicillin and 100 mg/ml streptomycin.

### Cell viability assay

Cells were seeded into 96-well plates. After attachment, cells were incubated with or without TSG (100 μM) for 24 h, and then incubated with or without APAP (0.48, 1.2, 3.0 mM) for another 48 h. After treatment, the viability of cells was detected by 3-(4,5-dimethyl-thiazol-2-yl) 2,5-diphenyltetrazolium bromide (MTT) method. Cell viability was calculated as the percentage of control.

### Real-time PCR analysis

Cells were seeded into 6-well plates. After attachment, cells were incubated with or without TSG (100 μM) for 6 h, 12 h, 18 h and 24 h, and then collected for the extraction of total RNA by using TRIZOL reagent. Liver total RNA was also extracted by using TRIZOL reagent. The quality and quantity of total RNA were determined by measuring the absorbance at wavelengths of the 260/280 nm ratio. The total RNA was reverse transcribed to cDNA and real-time RT-PCR analysis was conducted. Relative expression of genes was calculated by the 2^−△△Ct^ method and normalized to the house keeping gene β-actin, and given as ratio compared with the control. The primer sequences used in this study are shown in the supplementary Table.

### Protein extraction and Western-blot analysis

Cells were seeded into 6-well plates. After attachment, cells were incubated with or without TSG (100 μM) for 6 h, 12 h, 18 h and 24 h, and then collected for the extraction of proteins. Cellular proteins were isolated by using whole cell protein extraction kits. Cytosolic and nuclear proteins were isolated as described in NE-PER Nuclear and Cytoplasmic Extraction Reagents extraction kit. Liver tissues (50–100 mg) were homogenized in RIPA lysis buffer containing 1 mM phenylmethanesulfonyl fluoride (PMSF) and freshly prepared proteinase inhibitors. The supernatants were collected after the homogenates centrifuged at 15 000 × g for 10 min at 4 °C. Protein concentration was detected by BCA Kits, and all the samples in the same experiment were normalized to the equal protein concentration.

Protein samples was separated by SDS-PAGE gel and transferred into polyvinylidene difluoride membranes (PVDF). And the membranes were blocked with 5% dry non-fat milk in TBS containing 0.07% Tween-20 at room temperature for 1 h. The membranes were probed with appropriate combination of primary (overnight at 4 °C) and horseradish peroxidase-conjugated secondary antibodies. Protein-antibody complexes were visualized using an Enhanced Chemiluminescent reagent, and a ChemiDoc XRS system (Bio-Rad Laboratories, Inc., USA). Band intensities were semi-quantified by densitometry using Quantity One software (version 4.6.2, Bio-Rad Laboratories, Inc., USA). The protein bands were quantified by the average ratios of integral optic density following normalization to the expression of internal control actin, PCNA or Lamin B1, and the results were further normalized to control.

### Immunofluorescence staining

L-02 cells were seeded into dishes. After attachment, cells were incubated with or without TSG (100 μM) for 6 h, 12 h, 18 h and 24 h. After treatment, cells were fixed by 4% polyphosphate formaldehyde for 20 min, and then incubated with 0.3% triton X-100 for 10 min and further blocked with 1% bovine serum albumin (BSA) for 30 min. Cells were probed with appropriate combination of primary (overnight at 4 °C) and horseradish peroxidase- conjugated secondary antibodies. The first antibodies were incubated with cells overnight at 4 °C, and then cells were incubated with PE-CY5-conjugated secondary antibodies at 30 °C for 30 min avoiding light. 4′,6-Diamidino-2-phenylindole dihydrochloride (DAPI) was incubated with cells to stain the nucleus for 10 min. Immunofluorescence image analysis was implemented by live cell imaging system (Olympus, Japan).

### Statistical analysis

The software SPSS version 16.0 (SPSS, Inc., Chicago, IL, USA) was used for statistical analysis. Data were expressed as the means ± SEM. The significance of differences between groups was evaluated by one-way ANOVA with LSD post hoc test and *P* < 0.05 was considered as statistically significant differences.

## Electronic supplementary material

Supplementary Figure
