# Supplementary material for: 2,3,4′,5-tetrahydroxystilbene-2-O-β-D-glucoside exacerbates acetaminophen-induced hepatotoxicity by inducing hepatic expression of CYP2E1, CYP3A4 and CYP1A2
Source: Sci Rep. 2017 Nov 28;7:16511. doi: 10.1038/s41598-017-16688-5 (PMC5705655; doi:10.1038/s41598-017-16688-5)
Supplement: Supplementary file 1 — Supplementary Figure [file 41598_2017_16688_MOESM1_ESM.pdf]

**Manuscript title:** 2,3,4',5-tetrahydroxystilbene-2-O-β-D-glucoside exacerbates acetaminophen-induced hepatotoxicity by inducing hepatic expression of CYP2E1, CYP3A4 and CYP1A2

**Author List:** Shangfu Xu, Jie Liu, Jiangshan Shi, Zhengtao Wang, Lili Ji

**Supplementary Table: List of primer sequences used for real-time RT-PCR analysis.**

| Gene | Sequence (5'-3') | |
|---|---|---|
| | Forward primer | Reverse primer |
| Actb | CCTCTATGCCAACACGTGC | CCTGCTTGCTGATCCACATC |
| Cyp1a2 | ACAAGACCCAGAGCGAGAAG | GCAGCAGGATGGCTAAGAAG |
| Cyp2e1 | TGGGGAAACAGGGTAATGAG | GCACAGCCAATCAGAAAGGT |
| Cyp3a11 | AGAAGGCAAAGAAAGGCAAG | CTCTGGGTTGTTGAGGGAAT |
| ACTB | CATCCGCAAAGACCTGTACG | CCTGCTTGCTGATCCACATC |
| CYP1A2 | CCTCATCCTCCTGCTACCTG | CTGACACCACCACCTGATTG |
| CYP2E1 | CTCGTGGAAATGGAGAAGGA | TCAGAGTTGTGCTGGTGGTC |
| CYP3A4 | CCACTCACCCTGATGTCCA | AGCGTTTCATTCACCACCAT |

**Manuscript title:** 2,3,4',5-tetrahydroxystilbene-2-O-β-D-glucoside exacerbates acetaminophen-induced hepatotoxicity by inducing hepatic expression of CYP2E1, CYP3A4 and CYP1A2

**Author List:** Shangfu Xu, Jie Liu, Jiangshan Shi, Zhengtao Wang, Lili Ji

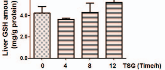


**Supplementary Figure 1.** TSG on liver GSH amount in mice. Liver GSH amount were determined after mice were treated with TSG (800 mg/kg) for 4 h, 8h or 12 h Data are shown as mean ± SEM (n=4).

**Manuscript title:** 2,3,4',5-tetrahydroxystilbene-2-O-β-D-glucoside exacerbates acetaminophen-induced hepatotoxicity by inducing hepatic expression of CYP2E1, CYP3A4 and CYP1A2

**Author List:** Shangfu Xu, Jie Liu, Jiangshan Shi, Zhengtao Wang, Lili Ji

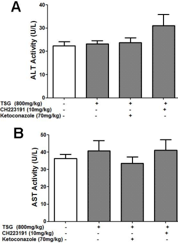


**Supplementary Figure 2.** Effects of TSG, ketoconazole or CH223191 on serum ALT and AST activity in mice. Serum ALT (A) and AST (B) activity was detected when mice orally given with TSG (800 mg/kg) for 12 h after pre-administrated with or without ketoconazole (70 mg/kg) or CH223191 (10 mg/kg) for 3 d (Once a day). Data are shown as mean ± SEM (n=5).

**Manuscript title:** 2,3,4',5-tetrahydroxystilbene-2-O-β-D-glucoside exacerbates acetaminophen-induced hepatotoxicity by inducing hepatic expression of CYP2E1, CYP3A4 and CYP1A2

**Author List:** Shangfu Xu, Jie Liu, Jiangshan Shi, Zhengtao Wang, Lili Ji

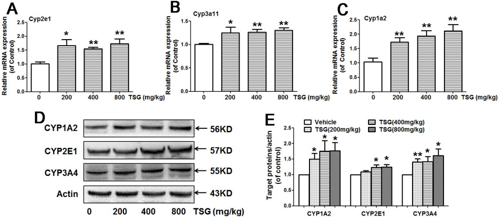


**Supplementary Figure 3.** TSG increased hepatic expression of CYP1A2, CYP2E1 and CYP3A4 in mice when C57BL/6 mice were treated with TSG for 5 consecutive days. (A) Hepatic mRNA expression of Cyp2e1 (n=5-7). (B) Hepatic mRNA expression of Cyp3a11 (n=5-7). (C) Hepatic mRNA expression of Cyp1a2 (n=5-8). (D-E) Hepatic protein expression of CYP1A2, CYP2E1 and CYP3A4. The results represent at least four independent experiments. Data were expressed as means ± SEM (n=4-8). *$P$<0.05, **$P$<0.01 compared to Vehicle control.
